# Effects of Repulsion Parameter and Chain Length of Homopolymers on Interfacial Properties of A*_n_*/A_x/2_B_x_A_x/2_/B*_m_* Blends: A DPD Simulation Study

**DOI:** 10.3390/polym13142333

**Published:** 2021-07-16

**Authors:** Dongmei Liu, Kai Gong, Ye Lin, Huifeng Bo, Tao Liu, Xiaozheng Duan

**Affiliations:** 1 School of Science, North China University of Science and Technology, Tangshan 063210, China; dmliu@ncst.edu.cn (D.L.); gongkai0524@163.com (K.G.); linye315317@163.com (Y.L.); 2 State Key Laboratory of Polymer Physics and Chemistry, Changchun Institute of Applied Chemistry, Chinese Academy of Sciences, Changchun 130022, China; 3 State Key Laboratory of Molecular Engineering of Polymers, Department of Macromolecular Science, Fudan University, Shanghai 200438, China

**Keywords:** dissipative particle dynamics, interfacial property, compatibilizer

## Abstract

We explored the effects of the repulsion parameter ($a_{AB}$) and chain length (*N*_HA_ or *N*_HB_) of homopolymers on the interfacial properties of A*_n_*/A_x/2_B_x_A_x/2_/B*_m_* ternary polymeric blends using dissipative particle dynamics (DPD) simulations. Our simulations show that: (i) The ternary blends exhibit the significant segregation at the repulsion parameter ($a_{AB}$ = 40). (ii) Both the interfacial tension and the density of triblock copolymer at the center of the interface increase to a plateau with increasing the homopolymer chain length, which indicates that the triblock copolymers with shorter chain length exhibit better performance as the compatibilizers for stabilizing the blends. (iii) For the case of *N*_HA_ = 4 (chain length of homopolymers A*_n_*) and *N*_HB_ (chain length of homopolymers B*_m_*) ranging from 16 to 64, the blends exhibit larger interfacial widths with a weakened correlation between bead A*_n_* and B*_m_* of homopolymers, which indicates that the triblock copolymer compatibilizers (A_x/2_B_x_A_x/2_) show better performance in reducing the interfacial tension. The effectiveness of triblock copolymer compatibilizers is, thus, controlled by the regulation of repulsion parameters and the homopolymer chain length. This work raises important considerations concerning the use of the triblock copolymer as compatibilizers in the immiscible homopolymer blend systems.

## 1. Introduction

Improving the performance of polymer materials in scientific and industrial applications can be achieved by mixing different components with complementary properties [1,2]. Typically, the mixing of two (or more) thermodynamically immiscible homopolymers (components) results in unstable interfaces within the systems and the poor mechanical properties of the blends [3]. For optimization, amphiphilic copolymers are often used as compatibilizers to improve the interfacial stability of highly immiscible mixtures in multi-constituent polymeric systems [4]. Specifically, the interfacially active amphiphilic copolymers preferentially aggregate at the interfaces between phase-separated homopolymers, which leads to a significant reduction in the interfacial tension between the two phases [5,6]. In consequence, the interfacial adhesion and mechanical properties of the blends can be improved by the significant increase in the thickness of the interface between the two phases during the mixing process [7,8,9].

In the past decades, considerable attention has been focused on the interfaces of binary and ternary blends [10,11,12,13,14,15,16,17,18,19,20,21,22,23,24,25,26,27,28,29]. For example, Qian et al. investigated the interfacial properties of the A*_n_*/B*_m_* binary homopolymers blends using DPD simulation [8]. It is found that when the chain length of the homopolymers was fixed, the interfacial tension increases with increasing Flory–Huggins interaction parameter; however, when the Flory–Huggins interaction parameter and the chain length of one homopolymer component were fixed, the interfacial tension increases slightly with increasing another homopolymer chain length. Guo et al. also employed DPD simulation to investigate the interfacial properties of A_2_/A_2_B_8_/B_2_ and A_2_/A_2_B_8_/B_10_ ternary mixtures [1]. They found that swelling is responsible for the stretching and orienting of the diblock copolymers chains and the reduced interfacial density of copolymers. The pioneering systematic experimental studies of homopolymer/triblock copolymer/homopolymer blend systems were performed by T. P. Russell et al. and E. J. Kramer et al. [22,23]. They initially reported that the triblock copolymers could serve as better compatibilizers than the diblock copolymers due to their higher critical micelle concentration for the same copolymer composition and concentration. Wolf et al. [24] investigated the poly(dimethyl siloxane)/poly(dimethyl siloxane)-poly(ethylene oxide)-poly(dimethyl siloxane) (PDMS/PDMS-PEO-PDMS/PEO) ternary blends. It was found that the interfacial tension increases with the rising of the temperature. Subsequently, through comparing the effects of the PDMS-PEO diblock copolymer compatibilizers and the PDMS-PEO-PDMS triblock copolymer compatibilizers on the interfacial tension of the PDMS/PEO blends, it was concluded that the interfacial tension of the blends mainly depended on the length of PDMS block, and has little correlation with the length of the PEO blocks and the compatibilizer architecture [25]. Xu et al. investigated the effects of Polymethyl methacrylate-polyethylene-Polymethyl methacrylate (PMMA-PE-PMMA) triblock copolymers on the properties of PE/PMMA blends. Their experiments showed that the PMMA-PE-PMMA triblock copolymers can significantly enhance the elastic modulus, the hardness, and the stability of the blends [26]. Sun et al. studied Poly(lactic acid)/poly(lactic acid)-poly(butylene adipate-*co*-terephthalate)poly(lactic acid)/poly(butylene adipate-*co*-terephthalate) (PLA/PLA-PBAT-PLA/PBAT) ternary blends, which showed that the addition of PLA-PBAT-PLA enhanced the miscibility and interfacial bonding strength between PLA/PBAT blends [27]. Recently, the interfacial tension of polypropylene/styrene-ethylene/polystyrene and polypropylene/butylene-styrene/polystyrene blends was investigated by Zhao et al [28], which indicated that the copolymers styrene–ethylene and butylene–styrene with a shorter chain length show higher efficiency than their long-chain length counterparts. In the previous study, we have investigated the dependence of the interfacial properties of symmetric ternary polymeric blends on the chain length and concentration of triblock copolymer, which also indicated that the triblock copolymers with shorter chain length perform a higher efficiency [29].

Despite the progress in the study of structural and thermodynamic properties of the blend systems, there are still many ambiguities in the A*_n_*/A_x/2_B_x_A_x/2_/B*_m_* ternary blends systems. For example, the study on the effect of repulsion parameters of different kinds of beads and the chain length of homopolymers on the ternary A*_n_*/A_x/2_B_x_A_x/2_/B*_m_* polymeric blends remains limited. In fact, the interfacial tension and the conformation of the ternary blends also depended on the repulsion parameter between the A and B beads, and on the chain length of homopolymers. It is necessary to clarify the effects of such factors on the structural and interfacial properties of the ternary blends.

In this work, we further use DPD simulation to explore the interfacial properties of symmetric ternary A*_n_*/A_x/2_B_x_A_x/2_/B*_m_* polymeric blends. We first briefly introduce the theory and algorithm of DPD simulations used in our work. We then systematically analyze the effects of repulsion parameters between beads A and B, and the chain length of the homopolymers on the properties of the interfaces in the blends, such as the interfacial tension, the density distribution of different beads and the detailed chain conformations of the triblock copolymers. Our work indicates that when the repulsion parameter is set to be $a_{AB}$ = 40, the ternary blends are significantly segregated and there exist flat interfaces between the two incompatible homopolymers. The triblock copolymers are more efficient in compatibilizing short incompatible homopolymers. Finally, we briefly summarize our results and offer some concluding remarks.

## 2. Methods

In a previous study, we employed the DPD method to investigate the effect of the chain length and concentration of triblock copolymer compatibilizers on the interfacial tension between two immiscible homopolymers [29]. The model of this work is constructed based on our previous work and other studies [8,9,29,30,31,32,33,34,35,36,37,38,39,40,41]. Herein, we briefly introduce the model; interested readers could refer to these studies for the further development of the model.

### 2.1. Model

We coarse-grain all the polymers as connected beads. The time evolution of the beads in the simulation satisfies Newton’s equations of motion,

(1) $$\frac{dr_{i}}{dt}=v_{i};\;m_{i}\frac{dv_{i}}{dt}=f_{i}$$

where $r_{i}$, $v_{i}$ and $m_{i}$ represent the position, velocity and mass of the *i*-th bead, respectively. The sum of conservative forces, dissipative forces, random forces and harmonic spring forces represents the total force $f_{i}$ acting on bead *i*, which can be expressed by the following formula [42]:

(2) $$f_{i}=\sum_{j\neq i}\left(F_{\mathrm{ij}}^{C}+F_{\mathrm{ij}}^{D}+F_{\mathrm{ij}}^{R}\right)+F_{i}^{S}$$

The conservative force $F_{\mathrm{ij}}^{C}$, dissipative force $F_{\mathrm{ij}}^{D}$ and random force $F_{\mathrm{ij}}^{R}$ are described as,

(3) $$F_{\mathrm{ij}}^{C}=-a_{AB}\omega^{C}\left(r_{\mathrm{ij}}\right)e_{\mathrm{ij}}$$

(4) $$F_{\mathrm{ij}}^{D}=-\gamma \omega^{D}\left(r_{\mathrm{ij}}\right)\left(v_{\mathrm{ij}}\cdot e_{\mathrm{ij}}\right)e_{\mathrm{ij}}$$

(5) $$F_{\mathrm{ij}}^{R}=\sigma \omega^{R}\left(r_{\mathrm{ij}}\right)\xi_{\mathrm{ij}}\cdot t^{-1/2}e_{\mathrm{ij}}$$

in which the repulsion force parameter $a_{AB}$ is a constant used to describe the repulsion between the different kinds of beads. $r_{\mathrm{ij}}=r_{i}-r_{j}$, $r_{\mathrm{ij}}=\left|r_{\mathrm{ij}}\right|$, $e_{\mathrm{ij}}=r_{\mathrm{ij}}/r_{\mathrm{ij}}$, and $v_{\mathrm{ij}}=v_{i}-v_{j}$. γ, σ and $\xi_{\mathrm{ij}}$ are interpreted as the friction coefficient, amplitude of the noise and Gaussian random number with zero mean and unit variance, respectively. $\omega^{C}$, $\omega^{D}$ and $\omega^{R}$ denote the three weight functions for the conservative, dissipative and random forces, respectively. For the conservative force $F_{\mathrm{ij}}^{C}$, we simply choose $\omega^{C}\left(r_{\mathrm{ij}}\right)=1-r_{\mathrm{ij}}$ for $r_{\mathrm{ij}}<1$ and $\omega^{C}\left(r_{\mathrm{ij}}\right)=0$ for $r_{\mathrm{ij}}\geq 1$. Unlike $\omega^{C}\left(r_{\mathrm{ij}}\right),$ $\omega^{D}\left(r_{\mathrm{ij}}\right)$ and $\omega^{R}\left(r_{\mathrm{ij}}\right)$ have a certain relation according to the fluctuation–dissipation theorem [31]:

(6) $$\omega^{D}\left(r\right)={\left[\omega^{R}\left(r\right)\right]}^{2},\;\sigma^{2}=2\gamma k_{B}T$$

where $k_{B}$ and *T* represent the Boltzmann constant and the temperature, respectively. The weight functions of dissipative and random forces are simply chosen as the previous work of Groot and Warren [42]:

(7) $$\omega^{D}\left(r\right)={\left[\omega^{R}\left(r\right)\right]}^{2}=\left\{\begin{array}{c} \begin{array}{cc} {\left(1-r\right)}^{2} & \left(r<1\right) \end{array}\\ \begin{array}{cc} 0 & \left(r\geq 1\right) \end{array} \end{array}\right.$$

The harmonic spring force $F_{i}^{S}$ is employed to account for the connection intrachain beads and can be expressed as

(8) $$F_{i}^{S}=\sum_{j}Cr_{\mathrm{ij}}$$

where *C* = 4.0 is the spring constant.

The conservative interaction strength between different types of beads $a_{AB}\;$(A and B are unlike beads) is chosen according to the linear relation with Flory–Huggins parameters χ for polymers [42]

(9) $$a_{AB}\approx a_{AA}+3.50\chi_{AB}$$

In this work, the repulsion parameter between the same type of beads is set as $a_{BB}$ = $a_{AA}$ = 25.

### 2.2. Simulation Details

We use the Materials Studio Program (Accelrys Inc.) to perform DPD simulations in a 30 × 30 × 30 simulation box with three-dimensional periodic boundary conditions. The radius of interaction, the bead mass and the temperature are set as $r_{c}=k_{B}T=1$ in the reduced unit (where $r_{c}$ is the interaction radius, *m* is the mass of bead, $k_{B}T$ is the temperature) according to the defaults of the program. The number density of the beads in the simulation system is fixed as ρ=3, and therefore, there are approximately 81,000 beads in each simulated system. The time step is taken as 0.05, and the friction coefficient γ is chosen as 4.5.

In this study, we focus on the ternary blends A*_n_*/A_2_B_4_A_2_/B*_m_* with the triblock copolymer concentration of *c*_cp_ = 0.05, 0.2 and the blends A*_n_*/A_7_B_14_A_7_/B*_m_* with *c*_cp_ = 0.2 (*c*_cp_ is referred as the number density of the triblock copolymer, *n* and *m* are the numbers of bead *A* and *B* of two immiscible homopolymers).

In order to examine the effects of the repulsion parameters $a_{AB}$ on interfacial properties, we vary $a_{AB}$ from 30 to 75. To investigate the chain length of homopolymers of interfacial properties, we change the chain length of homopolymers from *N*_H_ = 3 to 60 for the A*_n_*/A_2_B_4_A_2_/B*_m_* and A*_n_*/A_7_B_14_A_7_/B*_m_* (here the chain length of A*_n_* and B*_m_* are the same, hence, we use *N*_H_ to denote the chain length of homopolymers, i.e., *N*_H_ = *n* = *m*). In this study, the homopolymers and triblock copolymers are initially placed in the distinct parts in the box along the *x*-direction. These artificial initial configurations can speed up the formation of the interfaces perpendicular to the *x*-direction, so as to enhance the computing efficiency [8]. We first perform 2.0 × 10^5^ steps, which have proven to be sufficient to ensure that the system reaches an equilibrium state. To confirm the system equilibration, we calculate *R*_g_^2^ and *R*_ee_^2^ of copolymers as a function of the simulation time (as illustrated in Figure S1 in the supplementary material). Additionally, our previous work [29] and another previous study [43] also show that the relative systems could reach equilibration during such simulation time. Furthermore, we perform 5 × 10^4^ steps in the further production process. As shown in Figure S1 (t > 100,000 steps) in the supplementary material, although the fluctuation effects are not significant in our simulation, we still performed several parallel simulations and obtained final accurate results from the order of 10^3^ to 10^4^ independent statistical samples.

In a ternary blend with a flat interface, interfacial tension analysis can be used to detect the interface physical properties and stability of the blends. In addition, the interfacial tension results obtained from DPD simulations [33,34,35] are usually used as basic data to compare with Groot and Warren’s theoretical solutions. Here, we calculate the interfacial tension according to the Irving–Kirkwood equation [44]. The result is obtained by integrating the stress difference in the *x*-direction,

(10) $$\gamma_{\mathrm{DPD}}=\int \left[P_{\mathrm{xx}}-\frac{1}{2}\left(P_{\mathrm{yy}}+P_{\mathrm{zz}}\right)\right]dx$$

where *P* represents the pressure tensor, defined as the stress per unit area, *P*_xx_ represents the stress perpendicular to the interface and *P*_yy_ and *P*_zz_ represent the stress parallel to the interface.

We also calculate the mean-square radii of gyration <*R*_g_^2^>, mean-square end-to-end distance <*R*_ee_^2^> and the chain orientation parameter *q* of the triblock copolymers to characterize the detailed polymer conformations. We calculate the orientation parameters *q* according to the work of Qian et al. (Ref. [8]):

(11) $$q=\frac{\left(\langle R_{g}^{2}\rangle {}_{x}-1/2\left(\langle R_{g}^{2}\rangle {}_{y}+\langle R_{g}^{2}\rangle {}_{z}\right)\right)}{R_{g}^{2}}$$

where <*R*_g_^2^>_x_ is the normal component of the mean-square radii of gyration <*R*_g_^2^>, and <*R*_g_^2^>_y_ and <*R*_g_^2^>_z_ are the transverse components of mean-square radii of gyration <*R*_g_^2^>.

In addition, we calculate the interfacial width *w* between the immiscible homopolymers A*_n_* and B*_m_* according to the work of Guo et al. (Ref. [1]), the interfacial width *w* is obtained by fitting the function tanh ((*x + d*)/*w*) to the profile (ρ^A^(*x*)–ρ^B^(*x*))/ρ(*x*) across the two interfaces, where *d* is the shift of the interface center along with the *x* directions.

## 3. Results and Discussion

### 3.1. Effect of the Repulsion Parameters $a_{AB}$

**Figure 1:**
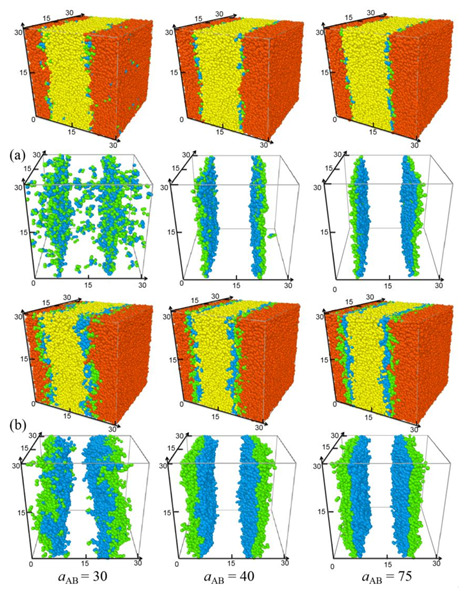
Morphology snapshots for ternary mixtures at different repulsion parameters $a_{AB}$ between A and B beads. The chain lengths and concentrations of the triblock are set as (**a**) A_2_B_4_A_2_, *c*_cp_ = 0.05 and (**b**) A_7_B_14_A_7_, *c*_cp_ = 0.2, respectively. The red and yellow spheres denote bead A and bead B of homopolymers A_8_ and B_8_, and the green and blue spheres represent beads A and B of the triblock.

**Figure 2:**
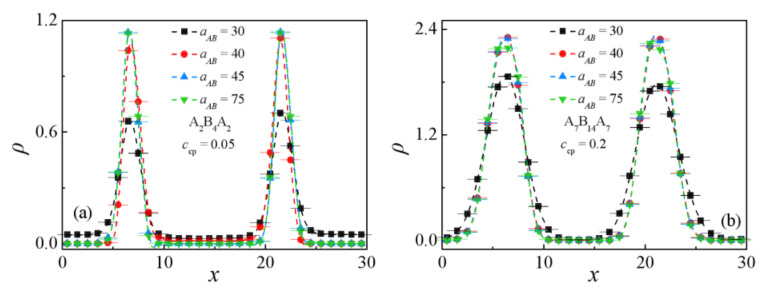
Density profiles of beads A + B of the triblock copolymer along the *x*-axis as a function of the repulsion parameter $a_{AB}$ with (**a**) A_2_B_4_A_2_, *c*_cp_ = 0.05 and (**b**) A_7_B_14_A_7_, *c*_cp_ = 0.2.

To fundamentally understand how the repulsion parameter influences the interfacial properties of homopolymer/triblock copolymer/homopolymer ternary blends, we fix the chain length of homopolymers *N*_H_ = 8 and vary the repulsion parameter $a_{AB}$ between beads A and B from 30 to 75 for the triblock copolymer A_2_B_4_A_2_ and A_7_B_14_A_7_ systems. Figure 1 and Figure 2 show the morphology snapshots and density profiles of triblock copolymers for the blend systems of A_8_/A_2_B_4_A_2_/B_8_ and A_8_/A_7_B_14_A_7_/B_8_, respectively. We found that most triblock copolymers are segregated at the interface, and the central B beads of the triblock copolymers preferentially segregate into the bulk phase of homopolymers B_8_, and both the end A beads segregate into the bulk phase of homopolymers A_8_, which indicates that the triblock copolymers form a “hairpin” type of conformation at the interfaces, as illustrated in Figure S2a. The structures of the copolymers result in the reduction of immiscible homopolymer contacts. Figure S2b in the supplementary material shows the morphology snapshot of the copolymer beads in blend A_8_/A_2_B_4_A_2_/B_8_ with $a_{AB}$ = 30, which illustrates that most triblock copolymers aggregate at the plane interface, and the rest of the copolymers aggregate into the homopolymer phase, as illustrated in Figure 2. We further consider the blends A_8_/A_2_B_4_A_2_/B_8_ with the triblock copolymer concentration of *c*_cp_ = 0.2. Figure S3 in the supplementary material shows the morphology snapshots of the blend. It can be seen that at such a condition, the interfaces have reached saturation, and the triblock copolymers A_2_B_4_A_2_ aggregate in a large amount in homopolymers A_8_, which could lead to the inaccurate calculation of the interfacial tension and the interfacial width. Moreover, in our previous work (ref. [29]), we found that the system A_8_/A_7_B_14_A_7_/B_8_ with *c*_cp_ = 0.05 exhibits larger interface tension, which indicates the instability of the interfaces. Due to the interface stability at the optimized triblock copolymer concentrations of *c*_cp_ = 0.05 for A*_n_*/A_2_B_4_A_2_/B*_m_* and *c*_cp_ = 0.2 for A*_n_*/A_7_B_14_A_7_/B*_m_*, we focus on these systems for the detailed analysis.

Figure 1 and Figure 2 show that the segregation of the triblock copolymer at the interface strongly depends upon the repulsion parameter $a_{AB}$. As the repulsion parameter $a_{AB}$ = 30, although the system shows an obvious interface and most of the triblock copolymers aggregate near the interface between homopolymer A_8_ and B_8_, the interface of the blend is not very smooth (see Figure 1a,b with $a_{AB}$ = 30) and the copolymers significantly penetrate the homopolymer bulk phase, as shown by the black solid squares in Figure 2a,b. As the repulsion parameter increases from $a_{AB}$ = 30 to 40, all of the triblock copolymers aggregate at the interface of the ternary blends (as shown by $a_{AB}$ = 40 in Figure 1), resulting in the increase in density of A + B beads of the triblock copolymers at the center of the interface (see the red solid dots in Figure 2a,b). However, as the repulsion parameter further increases from $a_{AB}$ = 40 to 75, the morphology and the density of the triblock copolymers change slightly. These findings indicate that as the repulsion parameter is set to be $a_{AB}$ = 40, the A_8_/A_2_B_4_A_2_/B_8_ and A_8_/A_7_B_14_A_7_/B_8_ ternary blends exhibit significant segregations, i.e., the two immiscible homopolymers are completely isolated by the copolymers, and almost all copolymers aggregate at the interface.

**Figure 3:**
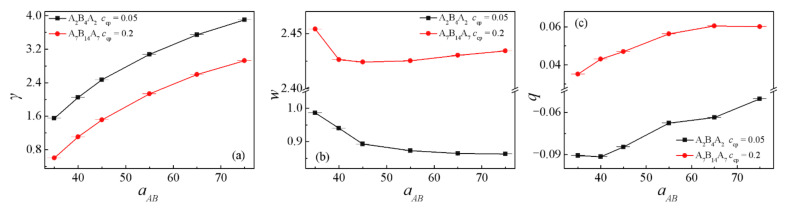
Interfacial tension $\gamma \;\left(a\right)$, interfacial thickness *w* (**b**) and orientation parameter (**c**) of the triblock as a function of repulsion parameters $a_{AB}$ between beads A and B.

**Figure 4:**
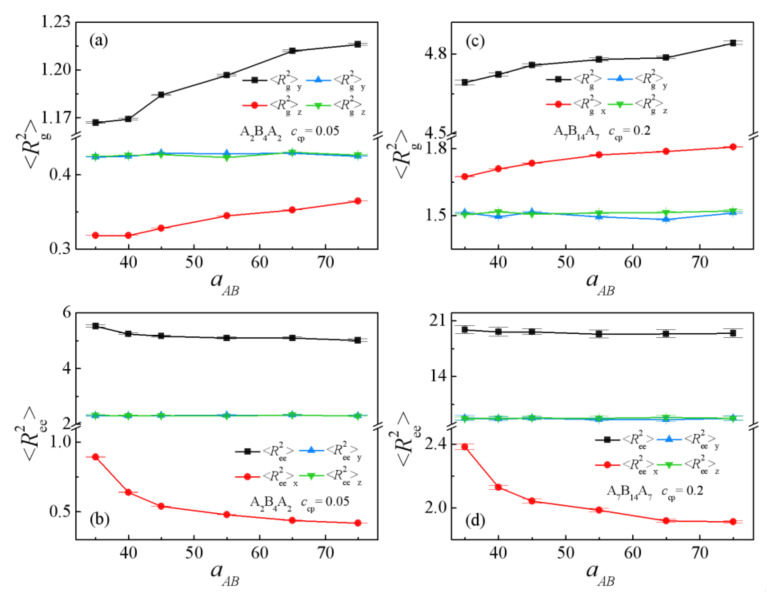
Mean-square radii of gyration <*R*_g_^2^> and the three components <*R*_g_^2^>_x_, <*R*_g_^2^>_y_, <*R*_g_^2^>_z_ as a function of the repulsion parameter $a_{AB}$ with (**a**) A_2_B_4_A_2_, *c*_cp_ = 0.05, (**c**) A_7_B_14_A_7_, *c*_cp_ = 0.2. Mean-square end-to-end distance <*R*_ee_^2^> and the three components <*R*_ee_^2^>_x_, <*R*_ee_^2^>_y_, <*R*_ee_^2^>_z_ as a function of the repulsion parameter $a_{AB}$ with (**b**) A_2_B_4_A_2_, *c*_cp_ = 0.05, (**d**) A_7_B_14_A_7_, *c*_cp_ = 0.2.

In order to quantitatively study the effects of the repulsion parameters $a_{AB}$ on the interfacial properties of the blend’s materials, we calculated the interfacial tension γ, the interfacial thickness *w*, the orientation parameter *q*, the mean-square radii of gyration <*R*_g_^2^> and the mean-square end-to-end distance <*R*_ee_^2^> at different repulsion parameter $a_{AB}$, as shown in Figure 3 and Figure 4. Apparently, the interfacial tension γ increases monotonically with increasing the repulsion parameter $a_{AB}$ for the two blend systems, as shown by Figure 3a. This finding is consistent with the simulations of the A*_n_*/B*_m_* binary blends of Qian et al. [8] and the A_2_/A_2_B_8_/B_2_(B_10_) ternary blends by Guo et al. [1]. Helfand and Tagami predicted the dependence of the interfacial tension γ on the interaction parameters χ by SCFT for the interface of homopolymer/homopolymer in the limit of infinite chain length, i.e., $\gamma =\rho bk_{B}T\sqrt{6\chi }$, which indicated that the interfacial tension is proportional to $\sqrt{6\chi }$ [45]. In our simulations, Figure 3a also shows a larger interfacial tension γ of blends at *c*_cp_ = 0.05. We inferred that the increase of the interfacial tension γ for this case with small copolymer concentration can be attributed to the distribution of copolymers beads, as illustrated in Figure 2a,b. It is shown that at the copolymer concentration *c*_cp_ = 0.05, the density of the beads A + B of the triblock copolymers exhibits a decrease, which results in the enhanced correlations between beads of immiscible homopolymers. Figure 3b shows the interfacial width *w* of the A_8_/A_2_B_4_A_2_/B_8_ and A_8_/A_7_B_14_A_7_/B_8_ system. For the A_8_/A_2_B_4_A_2_/B_8_ system, as the repulsion parameter increases from $a_{AB}$ = 35 to 55, the interfacial width *w* decreases, as the $a_{AB}$ increases further to 75, the interfacial width *w* of the changes slightly. In the A_8_/A_7_B_14_A_7_/B_8_ system, as the repulsion parameter increases from $a_{AB}$ = 35 to 75, the interfacial width *w* decrease first and then increases slowly. We inferred that the interface width *w*, which decreases first and changes slightly for the two cases with increasing the repulsion parameters $a_{AB}$, is related to the distribution of copolymers beads, i.e., as the density distribution of A + B beads of the triblock copolymer at the center of the interface is smaller, more A + B beads of the triblock copolymer penetrate the homopolymer phases, and this broader distribution of the triblock copolymer results in a larger interfacial width *w*.

Figure 3c and Figure 4 show the dependence of the chain orientation parameter *q* and the dimension (the mean-square radii of gyration <*R*_g_^2^> and its three components <*R*_g_^2^>_x_, <*R*_g_^2^>_y_, <*R*_g_^2^>_z_, the mean-square end-to-end distance <*R*_ee_^2^> and its three components <*R*_ee_^2^>_x_, <*R*_ee_^2^>_y_, <*R*_ee_^2^>_z_ of the copolymer, which can be used to characterize the detailed conformation of the copolymers at the interface) of the triblock copolymer on the repulsion parameter $a_{AB}$. It can be seen that the triblock chain orientation parameter *q* increases with increasing repulsion parameter $a_{AB}$; for the A_8_/A_2_B_4_A_2_/B_8_ system, *q* < 0 (the black solid square in Figure 3c), and for the A_8_/A_7_B_14_A_7_/B_8_ system, *q* > 0 (the red solid spheres in Figure 3c). Accordingly, Figure 4a,b show that <*R*_g_^2^> and <*R*_g_^2^>_x_ increase with increasing repulsion parameter $a_{AB}$, implying that the triblock copolymers are more stretched at larger repulsion parameters. In addition, we found that the <*R*_g_^2^>_y_ and <*R*_g_^2^>_z_ of triblock copolymers in *y* and *z* directions parallel to the interface are larger than the perpendicular <*R*_g_^2^>_x_ in the x-direction for the A_8_/A_2_B_4_A_2_/B_8_ system (Figure 4a), which is in contrast with the A_8_/A_7_B_14_A_7_/B_8_ system (Figure 4b). This finding is in agreement with the *q* of the two systems. Figure 4c,d show that the *x*-components <*R*_ee_^2^>_x_ of mean-square end-to-end distance decreases with increasing repulsion parameter $a_{AB}$, and the <*R*_ee_^2^>_y_ and <*R*_ee_^2^>_z_ are larger than <*R*_ee_^2^>_x_. These results indicated that as the repulsion parameter increase $a_{AB}$, the distribution of the end block A beads of the triblock copolymers is broader (as illustrated in Figure S4a,b) in the supplementary material, thus the *x* component of <*R*_ee_^2^>_x_ of the triblock copolymers is larger.

As shown by the previous studies, polymer blends with the greater interfacial tension exhibit the worse stability and adhesion of the interface [2]. Through comprehensive analysis for the interfacial tension γ of the blends, as well as the density distribution, chain orientation parameter *q* and dimension of the triblock copolymers, we conclude that as the repulsion parameter is $a_{AB}$ = 40, the ternary blends exhibit significant segregation with a lower interfacial tension and stronger adhesion. Therefore, in the following part of the manuscript, we fix the repulsion parameter of different types of beads as $a_{AB}$ = 40, and investigated the effect of chain length of homopolymers on the interfacial properties of A*_n_*/A_x/2_B_x_A_x/2_/B*_m_* polymeric blends.

### 3.2. Effect of Chain Length of Homopolymers

#### 3.2.1. Symmetric Homopolymers with N_HA_ = N_HB_

**Figure 5:**
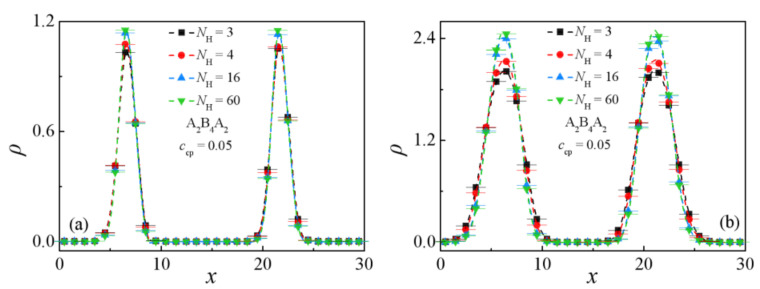
Density profiles of beads A + B of the triblock copolymer along the *x*-axis as a function of the homopolymer chain length with (**a**) A_2_B_4_A_2_, *c*_cp_ = 0.05, (**b**) A_7_B_14_A_7_, *c*_cp_ = 0.2.

We first consider the cases for homopolymers A*_n_* and B*_m_* with identical chain lengths, i.e., *N*_HA_ = *N*_HB_ = *N*_H_. Herein, the homopolymer chain length is varied from 3 to 60 for the triblock copolymer A_2_B_4_A_2_ and A_7_B_14_A_7_ ternary blends system. Figure 5 shows the relative density profiles of the triblock copolymers. It is shown that the segregation of the triblock copolymers at the interface depends on the chain length of homopolymers. The density of A + B beads of the triblock copolymer at the center of the interface increases with increasing the homopolymer chain length from *N*_H_ = 3 to *N*_H_ = 16. As the chain length of the homopolymer increases from *N*_H_ = 16 to *N*_H_ = 60, the density of A + B beads of the triblock copolymer at the center of the interface remains almost unchanged.

**Figure 6:**
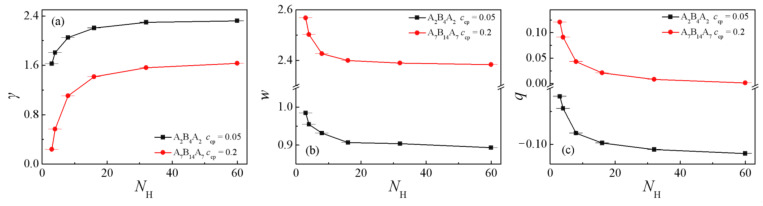
Interfacial tension $\gamma \;\left(a\right)$, interfacial thickness *w* (**b**), and orientation parameter *q* of the triblock (**c**) as a function of the homopolymer chain length *N*_H_ (*N*_H_ = 3, 4, 8, 16, 32, 48, 60).

Figure 6a,b show the dependence of the interfacial tension γ and the interfacial width *w* on the homopolymers chain length *N*_H_, respectively. The obtained interfacial tension γ rapidly increases and the interfacial width *w* decreases with increasing the homopolymer chain length from *N*_H_ = 3 to 32, whereas as *N*_H_ increases from 32 to 60, both the interfacial tension γ and the interfacial width *w* reach a plateau. These results show that: (i) the ternary blend system with shorter homopolymer chain length exhibits a lower interfacial tension γ, which implies that the triblock copolymers compatibilizers show better performance in reducing the interfacial tension of the ternary blends with shorter homopolymer chain length. This is because that the shorter the chain length of the homopolymers can cause a wider interfacial width *w* (Figure 6b), which results in the decayed correlations between beads of immiscible homopolymers and the smaller the interfacial tension γ.

**Figure 7:**
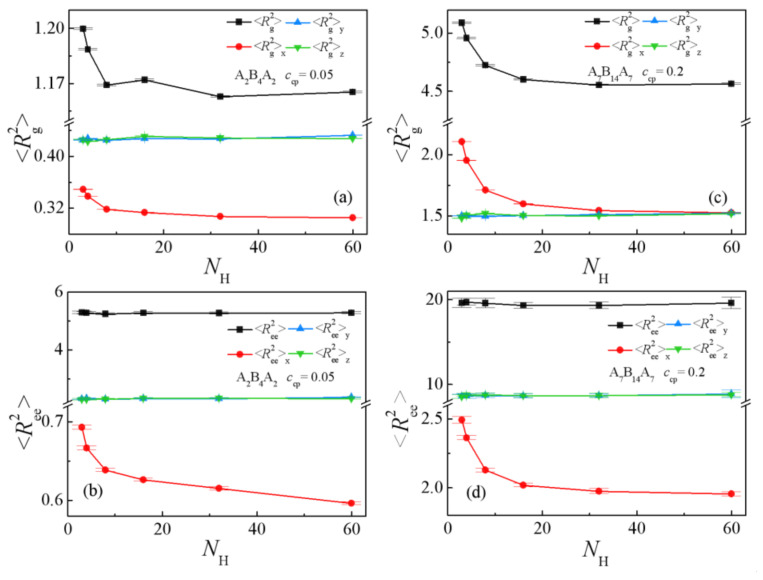
Mean-square radii of gyration <*R*_g_^2^> and the three components <*R*_g_^2^>_x_, <*R*_g_^2^>_y_, <*R*_g_^2^>_z_ as a function of the homopolymer chain length with (**a**) A_2_B_4_A_2_, *c*_cp_ = 0.05 (**c**) A_7_B_14_A_7_, *c*_cp_ = 0.2. Mean-square end-to-end distance <*R*_ee_^2^> and the three components <*R*_ee_^2^>_x_, <*R*_ee_^2^>_y_, <*R*_ee_^2^>_z_ as a function of the homopolymer chain length with (**b**) A_2_B_4_A_2_, *c*_cp_ = 0.05 (**d**) A_7_B_14_A_7_, *c*_cp_ = 0.2. (*N*_H_ = 3, 4, 8, 16, 32, 48, 60).

Figure 6c and Figure 7 show the chain orientation parameter *q* and the dimension of the triblock copolymers on the homopolymers chain length *N*_H_. We found that as the chain length of homopolymers increase from *N*_H_ =3 to 60, the chain orientation parameters *q* decrease first and then reach a plateau for the two systems (Figure 6c), which indicates that the triblock copolymers are more stretched at shorter homopolymer chain length in the *x*-direction, being perpendicular to the interface. Figure 7a,b show that <*R*_g_^2^>, <*R*_g_^2^>_x_, and <*R*_ee_^2^>_x_ of the copolymers decrease rapidly with the increasing of homopolymer chain length from *N*_H_ =3 to 8, whereas as *N*_H_ further increases from 8 to 60, only <*R*_ee_^2^>_x_ decreases slowly with the A_2_B_4_A_2_ system. For the A_7_B_14_A_7_ system, the <*R*_g_^2^>, <*R*_g_^2^>_x_ and <*R*_ee_^2^>_x_ decrease rapidly with the increase of homopolymer chain length from *N*_H_ = 3 to 16, but as *N*_H_ increases from 16 to 60, the <*R*_g_^2^>, <*R*_ee_^2^>_x_ and their three components almost unchanged (as illustrated in Figure 7c,d). We also found that as *N*_H_ increases, the *y* and *z* components (*y* and *z* are the directions parallel to the interface) of <*R*_g_^2^> and <*R*_ee_^2^> remain almost unchanged. The variation trend of the mean-square radii of gyration and the three components of the triblock copolymers corresponds well to the chain orientation parameter *q*. These results can be interpreted as follows: as the chain lengths of homopolymers are shorter, the triblock copolymers are more stretched in the *x*-direction, being perpendicular to the interface, and the distribution of the A end block of the triblock copolymers is broader (as illustrated in Figure S5a,b) in the supplementary material, which results in the larger values of *q*, <*R*_g_^2^>, <*R*_g_^2^>_x_ and <*R*_ee_^2^>_x_ of the triblock copolymers.

#### 3.2.2. The Chain Length Effect of Single Homopolymer Component

We further study the dependence of interfacial properties of the A*_n_*/A_7_B_14_A_7_/B*_m_* ternary blends on the chain length effect of single homopolymer component. For comparison, we consider the cases with (i) the chain length of polymer A*_n_* fixed as *N*_HA_ = 4, the chain length of polymer B*_m_* changing from *N*_HB_ = 4 to *N*_HB_ = 64, and (ii) *N*_HB_ = 4 fixed, *N*_HA_ changing from 4 to 64.

**Figure 8:**
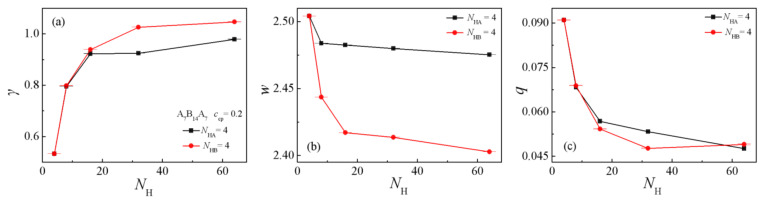
Interfacial tension $\gamma \;\left(a\right)$, interfacial thickness *w* (**b**), and orientation parameter *q* of the triblock (**c**) as a function of one kind homopolymer chain length *N*_H_ (*N*_H_ = 4, 8, 16, 32, 64).

Figure 8a,b show the dependence of the interfacial tension γ and the interfacial width *w* on the chain length of a single homopolymer component. As can be seen, the interfacial tension γ rapidly increases with increasing *N*_HA_ and *N*_HB_ from 4 to 32 at the two blend systems. This finding agrees well with the studies on A_2_/A_2_B_8_/B_2_(B_10_) ternary blends of Guo et al. [1]. When *N*_HA_ and *N*_HB_ increase from 32 to 60, the interfacial tension γ of the two systems remains almost unchanged, which is shown in Figure 8a. Figure 8b shows the interfacial width *w* for the two systems. For the case of *N*_HA_ = 4, the interfacial width *w* obviously decreases with increasing the homopolymer chain length *N*_HB_ from 4 to 8, whereas as *N*_HB_ increases from 8 to 64, the interfacial width *w* slowly decreases; for the system of *N*_HB_ = 4, the interfacial width *w* decreases with increasing of homopolymer chain length *N*_HA_ from 4 to 16, whereas as *N*_HA_ increases from 16 to 64, the interfacial width *w* slowly decreases. This result is because that the shorter the chain length of the homopolymers can cause a wider the interfacial width *w* (Figure 8b), which results in the decayed correlations between beads of immiscible homopolymers and the smaller interfacial tension γ.

**Figure 9:**
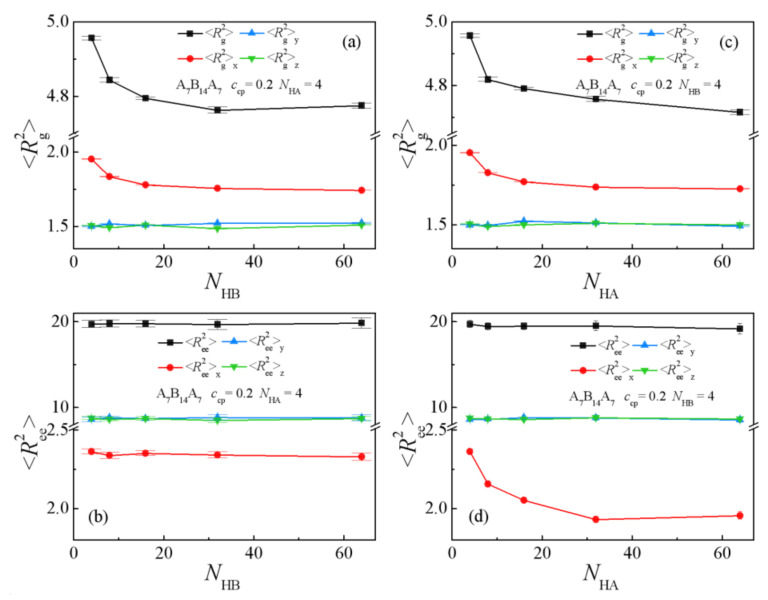
(**a**,**c**) Mean-square radii gyration <*R*_g_^2^> and the three components <*R*_g_^2^>_x_, <*R*_g_^2^>_y_, <*R*_g_^2^>_z_, (**b**,**d**) Mean-square end-to-end distance <*R*_ee_^2^> and the three components <*R*_ee_^2^>_x_, <*R*_ee_^2^>_y_, <*R*_ee_^2^>_z_ as a function of the chain length of single homopolymer component with A_7_B_14_A_7_, *c*_cp_ = 0.2. (*N*_HA_/*N*_HB_ = 4, 8, 16, 32, 60).

Figure 8c and Figure 9 show the chain orientation parameter *q* and the dimension of the triblock copolymers on the chain length of a single homopolymer component. The triblock chain orientation parameter *q* decreases with increasing homopolymer chain length *N*_HA_ and *N*_HB_ from 4 to 32, whereas as *N*_HA_ and *N*_HB_ further increase from 32 to 64, the *q* values of the triblock copolymers in both systems change slightly (as illustrated in Figure 8c). Figure 9 depicts the dependence of the dimension of the triblock copolymer on the chain length of a single component homopolymer. For the *N*_HA_ = 4 system (see the solid line in Figure 9a,b), the <*R*_g_^2^> and <*R*_g_^2^>_x_ of the triblock copolymers decrease with increasing homopolymers chain length *N*_HB_ from 4 to 16, whereas as *N*_HB_ increases from 16 to 64, the <*R*_g_^2^> and <*R*_g_^2^>_x_ remain almost unchanged. For the *N*_HB_ = 4 system (see the dotted line of Figure 9c,d), the variety of <*R*_g_^2^> and the three components are consistent with the *N*_HA_ = 4 system. Meanwhile, <*R*_ee_^2^>_x_ decreases rapidly with the increase of homopolymer chain length *N*_HA_ from 4 to 32, but as *N*_HA_ increases from 32 to 64, the <*R*_ee_^2^>_x_ almost remains unchanged. This implies that the increase of the homopolymer chain length *N*_HA_ has a greater impact on the <*R*_ee_^2^>_x_, which is directly related to the construction of the triblock copolymer, i.e., the two end blocks of the triblock copolymer composed of A beads. The results can be interpreted as follows: as the chain lengths of homopolymers A*_n_* are shorter and the chain length of homopolymers B*_m_* is fixed to be *N*_HB_ = 4, the distribution of the A end blocks of the triblock copolymers is broader, as illustrated in Figure S5d, which results in a larger value of <*R*_ee_^2^>_x_. By comparing Figure 9a–d with Figure 7c,d, we find that: (1) <*R*_g_^2^>_x_ is always larger than <*R*_g_^2^>_y_ or <*R*_g_^2^>_z_ for the *N*_HA_ = 4 and *N*_HB_ = 4 systems, whereas the three components of <*R*_g_^2^> are almost the same as the chain length of the homopolymers *N*_H_ > 32 for the *N*_HA_ = *N*_HB_ = *N*_H_ systems; (2) for the system with *N*_HA_ = 4, as *N*_HB_ increases from 4 to 64, the <*R*_ee_^2^>_x_ almost remains unchanged. These results illustrate that the stretch of the triblock copolymers depends on the chain length of the homopolymers, whereas <*R*_ee_^2^>_x_ only depends on the chain length of homopolymers *N*_HA_, due to the structure of the triblock copolymers. Specifically, the two end blocks (composed of *A* beads) of the triblock copolymers segregate into the homopolymer A*_n_* bulk phase (as illustrated in Figure 1). The increase in chain length of the homopolymer B*_m_* cannot significantly affect the structures of A end blocks of the copolymers, and therefore <*R*_ee_^2^>_x_, <*R*_ee_^2^>_y_, and <*R*_ee_^2^>_z_ almost remain unchanged.

The interfacial tension γ of the *N*_HA_ = 4 system (*N*_HB_ > 16) is less than the *N*_HB_ = 4 system (*N*_HA_ > 16); the interfacial width *w* of the *N*_HA_ = 4 system (*N*_HB_ > 16) is larger than the *N*_HB_ = 4 system (*N*_HA_ > 16), which results in the correlations between beads A*_n_* and B*_m_* of homopolymers decrease, thus the triblock copolymers compatibilizers show better performance in reducing the interfacial tension of the ternary blends with *N*_HA_ = 4.

The above analysis clearly shows that for the case of *N*_HA_ = *N*_HB_, *N*_HA_ = 4, and *N*_HB_ = 4, as the homopolymer chain length is *N*_H_ > 32, the interfacial properties slightly change with the increase of homopolymer chain length. This is because as the chain lengths of the homopolymers are much longer than the corresponding blocks of the triblock copolymers, the homopolymers cannot penetrate the copolymers blocks layer [20], and therefore the increase of the homopolymers chain length slightly affect the interfacial properties.

## 4. Conclusions

In this paper, we investigated the effects of the repulsion parameter and chain length of the homopolymers on the interfacial properties of ternary A*_n_*/A_x/2_B_x_A_x/2_/B*_m_* polymeric blends using dissipative particle dynamics (DPD) simulations.

By comparing the interfacial tension and the density distribution of the triblock copolymers of the A_8_/A_2_B_4_A_2_/B_8_ and A_8_/A_7_B_14_A_7_/B_8_ blends at different repulsion parameters, we find that at a repulsion parameter ($a_{AB}$ = 40), the ternary blends exhibit the maximum segregation with lower interfacial tension and stronger adhesion.

We then compare the efficiency of the triblock copolymer on stabilizing the blends of incompatible homopolymers at different chain lengths. For the case of chain lengths of homopolymers *N*_HA_ = *N*_HB_, both the interfacial tension and the density of triblock copolymer at the center of the interface increase to a plateau with increasing the homopolymer chain length, which indicates that the triblock copolymers exhibit better performance as the compatibilizers for blending homopolymers with shorter chain length due to the more stretched conformations of triblock copolymers. For the case of fixing one homopolymer chain length system (the system with *N*_HA_ = 4 or the system with *N*_HB_ = 4), the triblock copolymers (A_x/2_B_x_A_x/2_) compatibilizers show better performance in reducing the interfacial tension for blends with *N*_HA_ = 4, compared to the blends with *N*_HB_ = 4.

Our simulations indicate that the interfacial properties of the ternary A*_n_*/A_x/2_B_x_A_x/2_/B*_m_* polymeric blends are strongly correlated to repulsion parameters and the chain length of the incompatible homopolymers, which provides insights into the fundamental understanding of the interfacial properties of polymer blends. In this context, it would also be interesting to systematically study the influence of other physical factors on the interfacial properties of the polymeric blend at the microscopic level. In addition, with the development of polymer synthesis technology, ionic polymer blends have attracted extensive attention as new materials due to their ideal ionic conductivity and mechanical strength [46]. We predict that adding block copolymers (as a compatibilizer) into the ionic polymeric blends might modify the physicochemical property of the polymer electrolytes.

## Data Availability

Data is contained within the article or supplementary material.

## Supplementary Materials

The following are available online at https://www.mdpi.com/article/10.3390/polym13142333/s1, Figure S1: (a) *R*_g_^2^ and (b) *R*_ee_^2^ of the triblock copolymers for the case A_8_/A_2_B_4_A_2_/B_8_ with *c*_cp_=0.05 as a function of the simulation time; (c) *R*_g_^2^ and (d) *R*_ee_^2^ of the triblock copolymers for the case A_8_/A_7_B_14_A_7_/B_8_ with *c*_cp_ = 0.2 as a function of the simulation time. Figure S2: (a) Representative snapshots of the “hairpin” structure for A_7_B_14_A_7_. (b) Morphology snapshot of the copolymers for A_2_B_4_A_2_, *c*_cp_ = 0.05, $a_{AB}$ = 30. The red and yellow spheres denote bead A and bead B of homopolymers A_8_ and B_8_, and the green and blue spheres represent beads A and B of the triblock. Figure S3: Morphology snapshots for ternary mixtures A_8_/A_2_B_4_A_2_/B_8_, *c*_cp_ = 0.2, $a_{AB}\;$= 40. The red and yellow spheres denote bead A and bead B of homopolymers, and the green and blue spheres represent beads A and B of the triblock., Figure S4: Density profiles of beads A, B of the triblock copolymer along the *x*-axis as a function of the repulsion parameter $a_{AB}$ with (a) A_2_B_4_A_2_, *c*_cp_ = 0.05 and (b) A_7_B_14_A_7_, *c*_cp_ = 0.2. Figure S5: Density profiles of beads A, B of the triblock copolymer along the *x*-axis as a function of chain length of the homopolymers *N*_H_ = *N*_HA_ = *N*_HB_ with (a) A_2_B_4_A_2_, *c*_cp_ = 0.05, (b) A_7_B_14_A_7_, *c*_cp_ = 0.2. Density profiles of beads A, B of the triblock copolymer along the *x*-axis as a function of one homopolymers chain length with (c) A_7_B_14_A_7_, *c*_cp_ = 0.2, *N*_HA_ = 4 (d) A_7_B_14_A_7_, *c*_cp_ = 0.2, *N*_HA_ = 4.
