# Supplementary material for: Effects of Repulsion Parameter and Chain Length of Homopolymers on Interfacial Properties of An/Ax/2BxAx/2/Bm Blends: A DPD Simulation Study
Source: Polymers (Basel). 2021 Jul 16;13(14):2333. doi: 10.3390/polym13142333 (PMC8309644; doi:10.3390/polym13142333)
Supplement: Supplementary file 1 [file polymers-13-02333-s001.zip › polymers-1276224-supplementary.pdf]

polymers

MDPI

Article

Effects of Repulsion Parameter and Chain Length of Homopolymers on Interfacial Properties of $A_n/A_{x/2}B_xA_{x/2}/B_m$ Blends: A DPD Simulation Study

Dongmei Liu [1], Kai Gong [1], Ye Lin [1], Huifeng Bo [1,*], Tao Liu [1,*] and Xiaozheng Duan [2,3,*]

1 School of Science, North China University of Science and Technology, Tangshan 063210, China; dmliu@ncst.edu.cn (D.L.); gongkai0524@163.com (K.G.); linye315317@163.com (Y.L.)

2 State Key Laboratory of Polymer Physics and Chemistry, Changchun Institute of Applied Chemistry, Chinese Academy of Sciences, Changchun 130022, China

3 State Key Laboratory of Molecular Engineering of Polymers, Department of Macromolecular Science, Fudan University, Shanghai 200438, China

* Correspondence: bohuifeng@ncst.edu.cn (H.B.); liutao@ncst.edu.cn (T.L.); xzduan@ciac.ac.cn (X.D.); Tel.: +86-315-8805860 (H.B. & T.L.); +86-431-85262479 (X.D.)

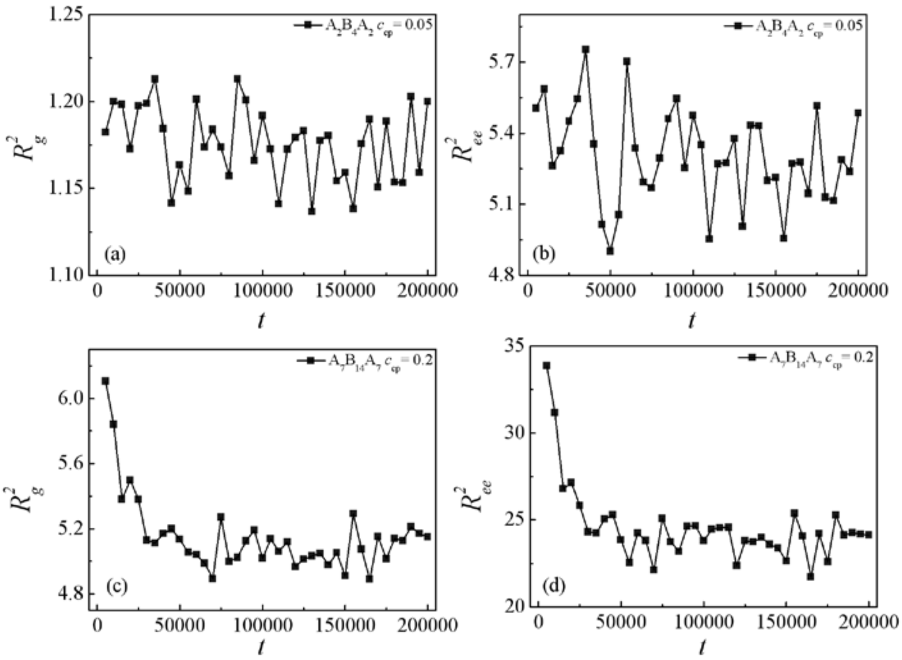

Figure S1. (a) R_g^2 and (b) R_{ee}^2 of the triblock copolymers for the case $A_8/A_2B_4A_2/B_8$ with c_{cp}=0.05 as a function of the simulation time; (c) R_g^2 and (d) R_{ee}^2 of the triblock copolymers for the case $A_8/A_7B_{14}A_7/B_8$ with c_{cp}=0.2 as a function of the simulation time.

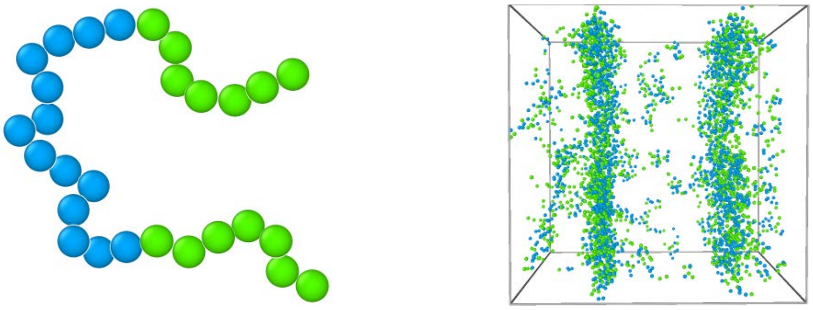

(a) (b)

Figure S2. (a) Representative snapshots of the "hairpin" structure for $A_7B_{14}A_7$. (b) Morphology snapshot of the copolymers for $A_2B_4A_2$, c_{cp} = 0.05, a_{AB} = 30. The red and yellow spheres denote bead A and bead B of homopolymers A_8 and B_8, and the green and blue spheres represent beads A and B of the triblock.

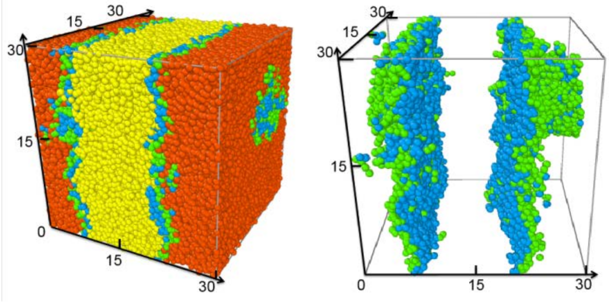

Figure S3. Morphology snapshots for ternary mixtures $A_8/A_2B_4A_2/B_8$, c_{cp} = 0.2, a_{AB}= 40. The red and yellow spheres denote bead A and bead B of homopolymers, and the green and blue spheres represent beads A and B of the triblock.

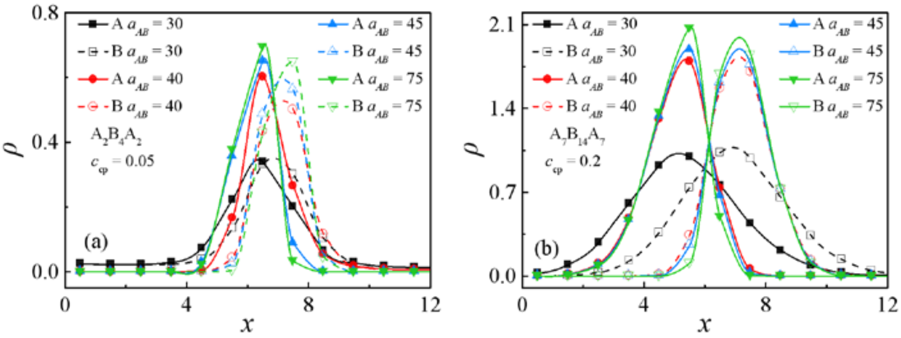

Figure S4. Density profiles of beads A, B of the triblock copolymer along the x-axis as a function of the repulsion parameter a_{AB} with (a) $A_2B_4A_2$, c_{cp} = 0.05 and (b) $A_7B_{14}A_7$, c_{cp} = 0.2.

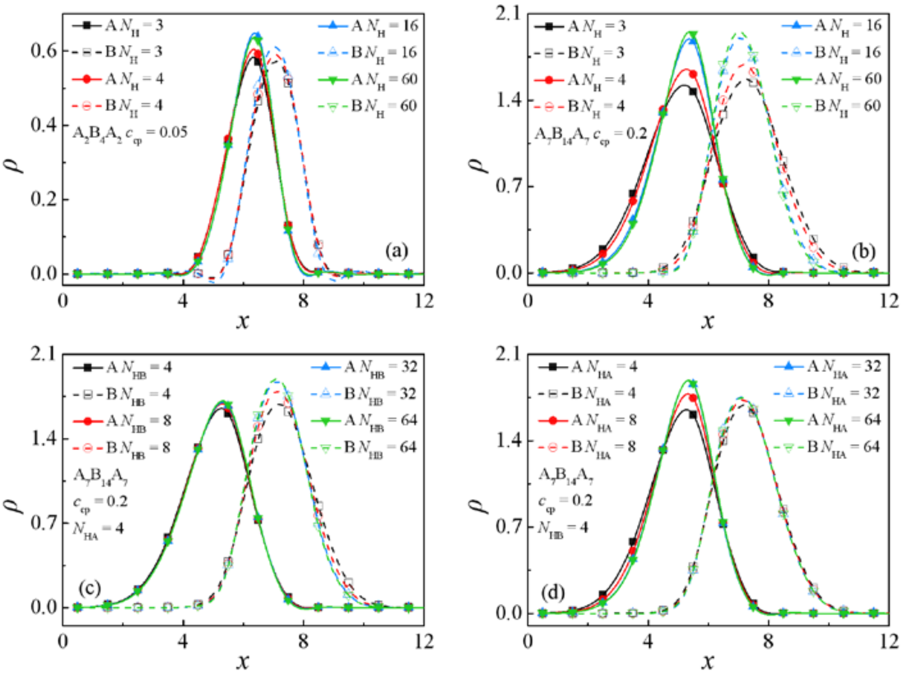

Figure S5. Density profiles of beads A, B of the triblock copolymer along the x-axis as a function of chain length of the homopolymers N_H = N_{HA} =N_{HB} with (a) $A_2B_4A_2$, c_{cp} = 0.05, (b) $A_7B_{14}A_7$, c_{cp} = 0.2. Density profiles of beads *A*, *B* of the triblock copolymer along the x-axis as a function of one homopolymers chain length with (c) $A_7B_{14}A_7$, c_{cp} = 0.2, N_{HA} = 4 (d) $A_7B_{14}A_7$, c_{cp} = 0.2, N_{HA} = 4.
